# 

**Published:** 1916-06-10

**Authors:** 


### THE KING'S BIRTHDAY HONOURS.

### Naval and Military Lists.

THE following lists of medical officers decorated or otherwise rewarded for naval and military services are extracted from the general lists issued in the *London Gazette* and Supplement on the occasion of the King's birthday, and from a short list which came out two or three days earlier. There are also over 500 awards of the Royal Red Cross, of the First and Second Classes:—

C.B.

Surg.-Gen. W. G. A. Bedford, C.M.G.
Surg.-Gen. R. Porter.
Surg.-Gen. T. J. O'Donnell, D.S.O.
Col. R. H. Luce, F.R.C.S., T.F. Reserve.
Lieut.-Col. C. C. Cumming, R.A.M.C.
Fleet-Surg. E. C. Lomas, D.S.O., R.N.
Surg.-Gen. J. J. Dennis, M.D., R.N.
Col. A. P. Blenkinsop, A.D.G., A.M.S.
Major P. S. Lelean, R.A.M.C.

K.C.M.G.

Col. (Hon. Surg.-Gen.) W. D. C. Williams, C.B., Australian A.M.C.

C.M.G.

Col. C. E. Harrison, C.V.O., F.R.C.S., A.M.S. T.F.
Col. H. N. Thompson, D.S.O., A.M.S.
Col. T. B. Beach, A.M.S.
Col. C. W. R. Bealey, A.M.S.
Col. J. B. Wilson, A.M.S.
Col. A. L. F. Bate, A.M.S.
Col. E. C. Freeman, A.M.S. T.F.
Temp. Col. A. Fullerton, A.M.S.
Lieut.-Col. H. E. R. James, C.B., F.R.C.S., R.A.M.C.
Lieut.-Col. A. H. Lister, F.R.C.S., R.A.M.C. T.F.
Lieut.-Col. O. L. Robinson, R.A.M.C.
Lieut.-Col. H. A. Bray, R.A.M.C.
Lieut.-Col. (temp. Col.) E. W. Slayter, R.A.M.C.
Lieut.-Col. T. P. Jones, R.A.M.C.
Lieut.-Col. G. S. Thom, R.A.M.C.
Lieut.-Col. J. V. Forrest, R.A.M.C.
Lieut.-Col. L. Humphrey, R.A.M.C.
Temp. Lieut.-Col. W. H. Willcox, M.D., F.R.C.P., R.A.M.C.
Lieut.-Col. (Hon. Col.) Sir J. R. A. Clark, Bt., C.B., F.R.C.S., R.A.M.C. T.F.
Major G. Hall, M.D., R.A.M.C. T.F.
Lieut.-Col. B. J. Newmarch, Australian A.M.C.
Lieut.-Col. (temp. Col.) J. T. Fotheringham, Australian A.M.C.
Lieut.-Col. P. C. Fenwick, New Zealand M.C.

C.I.E.

Lieut.-Col. J. Jackson, I.M.S.
Major F. N. White, M.D., I.M.S.
Capt. A. G. J. MacIlwaine, R.A.M.C.

D.S.O.

Major H. V. Bagshawe, R.A.M.C.
Major B. S. Bartlett, R.A.M.C.
Lieut.-Col. E. A. Bourke, R.A.M.C.
Surg.-Major R. M. Cowie, 1st Life Guards.
Major J. M. Darling, R.A.M.C. Sp. Res.
Major A. N. Fraser, R.A.M.C.
Capt. E. D. Gairdner, R.A.M.C. T.F.
Lieut.-Col. J. S. Gallie, R.A.M.C.
Lieut.-Col. J. Grech, R.A.M.C.
Major P. H. Henderson, R.A.M.C.
Major G. W. Heron, R.A.M.C.
Capt. A. H. Heslop, R.A.M.C.
Capt. G. S. Husband, I.M.S.
Temp. Capt. J. L. Ingram, R.A.M.C.
Major (temp. Lieut.-Col.) A. C. Osburn, R.A.M.C.
Lieut.-Col. E. P. Sewell, R.A.M.C.
Capt. W. C. Smales, R.A.M.C.
Major W. M. B. Sparkes, R.A.M.C.
Major C. P. Thomson, R.A.M.C.
Capt. (temp. Major) A. E. Webb-Johnson, F.R.C.S., R.A.M.C. T.F.
Major J. B. Maclean, Australian A.M.C.
Capt. T. F. Brown, Australian A.M.C.
Capt. (temp. Major) R. D. Campbell, Australian A.M.C.

MILITARY CROSS.

Temp. Capt. G. S. Blandy, M.D., R.A.M.C.
Capt. J. C. Brash, R.A.M.C.
Capt. W. A. Brechin, R.A.M.C. T.F.
Temp. Capt. A. Bremner, R.A.M.C.
Capt. L. C. Bruce, M.D., F.R.C.P., R.A.M.C. T.F.
Temp. Capt. W. H. Butler, R.A.M.C.
Temp. Lieut. J. W. Corkey, R.A.M.C.
Capt. W. R. Douglas, R.A.M.C. T.F.
Capt. K. K. Drury, R.A.M.C. Sp. Res.
Temp. Capt. C. R. Dudgeon, R.A.M.C.
Capt. G. F. Dyass, R.A.M.C.
Temp. Capt. A. C. Edwards, R.A.M.C.
Capt. T. J. C. Evans, F.R.C.S., I.M.S.
Capt. J. F. Farrow, R.A.M.C. T.F.
Capt. A. D. Fraser, R.A.M.C.
Temp. Lieut. C. T. Galbraith, R.A.M.C.
Capt. J. Gilmour, F.R.C.S., R.A.M.C.
Capt. G. R. Grant, R.A.M.C. Sp. Res.
Temp. Capt. E. R. G. Greville, R.A.M.C.
Capt. H. P. Hart, R.A.M.C.
Qmr. and Hon. Lieut. C. F. Houston, R.A.M.C.
Temp. Capt. A. C. Keep, M.D., R.A.M.C.
Temp. Capt. A. J. Kendrew, R.A.M.C.
Temp. Lieut. W. J. Knight, R.A.M.C.
Capt. C. Lovell, M.D., R.A.M.C.
Temp. Capt. M. A. Macdonald, R.A.M.C.
Temp. Lieut. J. S. McCallam, M.D., R.A.M.C.
Capt. F. A. McCammon, R.A.M.C.
Capt. J. D. MacCormack, R.A.M.C. Sp. Res.
Temp. Lieut. P. McGibbon, R.A.M.C.
Capt. H. S. Milne, M.D., R.A.M.C.
Temp. Lieut. J. W. O'Brien, R.A.M.C.
Temp. Capt. H. J. Orr-Ewing, R.A.M.C.
Capt. H. F. Panton, R.A.M.C.
Capt. D'Arcy Power, R.A.M.C. Sp. Res.
Capt. G. E. J. A. Robinson, M.D., R.A.M.C. T.F.
Surg.-Capt. W. T. Rowe, M.D., Notts Yeo.
Temp. Capt. H. H. Sampson, F.R.C.S., R.A.M.C.
Capt. L. R. Shore, R.A.M.C.
Lieut. C. J. Stocker, I.M.S.
Temp. Capt. M. A. Swan, R.A.M.C.
Capt. D. C. Taylor, F.R.C.S., R.A.M.C.
Temp. Lieut. N. S. Whitton, R.A.M.C.
Capt. H. F. Wilkins, F.R.C.S., R.A.M.C. T.F.
Temp. Capt. P. R. Woodhouse, R.A.M.C.
Capt. G. E. Kidd, Canadian A.M.C.

TO BE HONORARY CAPTAIN.

Qmr. and Hon. Lieut. E. W. Newland, R.A.M.C.

TO HIGHER RATES OF PAY.

Qmr. and Hon. Lieut. E. Bennett, R.A.M.C.
Qmr. and Hon. Lieut. G. A. Collier, R.A.M.C.

### Buildings Contemplated.

BULKINGTON.—The Sanitary Committee of the Coventry Corporation have approved the draft agreement of tenancy of the Bramcote Hospital, in the parish of Bulkington, which the Warwickshire and Coventry Joint Committee for Tuberculosis are desirous of taking for a sanatorium at a rent of £350 per annum.

HEREFORD.—The Governors of the Herefordshire General Hospital have decided to earmark Miss Norton's legacy of £500 for the proposed Nurses' Home.

MORTON BANKS.—An opportunity of inspecting the plans for the extension of the Morton Banks Hospital in connection with the military hospital scheme has been afforded the Keighley Rural District Council. Two hundred beds will be provided in the existing buildings, and an additional 200 in the new buildings.

NUNEATON.—Weddington Hall, a Warwickshire mansion on the outskirts of Nuneaton, is to be converted into a hospital for wounded soldiers.

SWANSEA.—The Town Council have received sanction from the Local Government Board to borrow £5,000 to build and equip a small-pox hospital.

### MUNIFICENT GIFT TO CARDIFF HOSPITAL.

IN addition to the special donations announced last week, King Edward VII.'s Hospital has received from Lieut.-Commander Sir Edward Nicholl the sum of £10,000. This is also for the proposed extensions, which are now assured of completion. This generous donor is one of Cardiff's most poular shipowners, and received the honour of knighthood from His Majesty on Saturday last. With its proposed extensions, the Cardiff Hospital will take a leading place in the kingdom, both as regards accommodation and equipment. It is to be hoped that Sir Edward Nicholl's example will be widely followed, so that the scheme may be completed without delay.

### The Book Market.

LIST OF NEW BOOKS RECEIVED FROM THE FOLLOWING PUBLISHERS:—

Burns and Oates, Ltd.: "Who Goes There?" By the Author of "Aunt Sarah and the War." 1s. net.

Kegan Paul, Trench, Trübner and Co., Ltd.: "Nervous Diseases of Women." By Bernard Hollander, M.D. 3s. 6d. net.

Macmillan and Co.: "The Cancer Problem." By W. S. Bainbridge, A.M., Sc.D., M.D. 17s. net. "The New Public Health." By H. Winslow Hill, M.B., M.D., D.P.H. 5s. 6d. net. "Under Three Flags: With the Red Cross in Belgium, France, and Serbia." 3s. 6d. net.

### PASSING EVENTS AND TOPICS.

### Scottish Help for the French Red Cross.

At a floral fête and sale organised by the Countess of Elgin, and held at Dunfermline, on behalf of the French Red Cross, the sum collected amounted to nearly £600.

### A Handsome Gift.

Sir William Milligan has offered to the Manchester Royal Infirmary twelve machines of the "Zander" type for the active treatment of stiff joints and immobile limbs as a nucleus of an orthopædic department.

### Patients' Roll Call at St. Bartholomew's.

The annual "view" of the patients at St. Bartholomew's Hospital was held on May 10, when Lord Sandhurst, accompanied by the almoners, governors, the clerk, the matron, and the stewards, visited every ward and department. The steward called out the names of the patients, among whom there were 200 wounded soldiers. Prizes were presented to sisters and nurses whose wards have been in the most satisfactory state during the past year.

### The Scottish Women's Hospital.

The London correspondent of the *Glasgow Herald* states that Miss Kathleen Burke, the London secretary of the Scottish Women's Hospitals, who is now in America raising money for the hospitals, has sent home some four thousand pounds as the result of two weeks' work.

### New Tuberculosis Hospital at Newport.

The Welsh Insurance Commissioners have approved Cardigan House, Newport, as a tuberculosis hospital for the period of the war. This house was presented to the Welsh National Memorial Association by Sir Garrod Thomas, and a donation of £1,000 by Messrs. Griffiths Brothers, of Newport, was also given to the Association for the purpose of equipping it. The hospital is expected to be ready for occupation by the end of July, when twenty-four beds will be available for crippled children. Major Morrell Thomas, M.D., F.R.C.S., has been appointed consulting surgeon and Dr. T. A. Lee aural surgeon.

### HOSPITAL RESULTS IN 1915.

**British Home for Mothers and Babies, Woolwich.**—This is the first annual report of the institution formed by the amalgamation in 1915 of the British Lying-in Hospital (late in Endell Street), founded in 1749, and the Home for Mothers and Babies, founded in 1905. The scheme included the building of a new hospital in Woolwich, which is apparently at the present time one of the most unfavourable places in England for building operations on account of the scarcity of labour, the Government having undertaken the erection of 3,000 houses for the accommodation of Arsenal workers. The extra cost of building material (over 30 per cent.) is another reason for delay. This is a pity, as there is obviously plenty of work in this crowded locality to be done by a lying-in hospital which also concerns itself with the after-care of the mothers and babies. But with the accommodation at its disposal work has proceeded with much vigour, and we note from the accounts that, although more help is needed towards the building scheme, the current operations are carried on without any burden of debt.

**Glasgow Royal Maternity and Women's Hospital.**—The total receipts for 1915 were £9,360, and the total expenditure £9,357, a state of affairs which would have delighted Mr. Micawber. The hospital has ante-natal and post-natal departments, and an almoner has been appointed. Thirty-nine Belgian refugees were treated during the year.

**London Fever Hospital.**—The average weekly cost of each in-patient has increased from £2 13s. 7d. in 1914 to £3 4s. in 1915. The total expenditure exceeds the total income by £1,084. Annual subscriptions show a decrease of £117, but donations increased by £550. There were 200 fewer cases of scarlet fever treated as compared with 1914, but over 300 more patients suffering from English and German measles. The death-rate from scarlet fever, as shown by the hospital records, has fallen from 14.2 per cent. in the period 1860-1864 to 1.2 per cent. in the period 1910-1914, and from diphtheria from 23 per cent. (1880-1884) to 3.5 per cent. (1910-1914). Fees for private rooms have been increased to 5½ guineas per week, and ward fees for diphtheria to 3½ guineas per patient.

**National Hospital for the Paralysed and Epileptic.**—The report for 1915 shows that the income of the hospital and its country convalescent branch increased from £22,101 to £24,373, and the expenditure from £19,021 to £23,708. The receipts under all headings were well maintained; patients' payments amounted to £6,974, which amount includes £2,384 received in respect of the sixty-eight beds allocated to military cases. There was a large increase in the cost of drugs, mainly due to the phenomenal prices of bromides, which are chiefly used for the treatment of epilepsy. The massage school and school of electrical treatment appear to be features of growing importance.

**Royal West Sussex Hospital, Chichester.**—The expense of producing an annual report in this case has been substantially met by eleven pages of advertisements, but the cost of postage must have been added to by the use of unnecessarily heavy paper. Two hundred and fifty-eight sick and wounded soldiers were admitted in 1915. The income exceeded the expenditure by £1,011; no legacies available for current expenditure were received, but there was an increase in the amount obtained from annual subscriptions. A Ladies' Linen League contributed 1,545 articles.

**South Devon and East Cornwall Hospital, Plymouth.**—The number of in-patients treated in 1915 (1,729) was in excess of any year since the charity was founded, although the average stay was longer than in any year since 1909. The beds for wounded soldiers have been increased from fifty to sixty. A new mortuary chapel has been completed, and many special donations have been given towards this particular object. Ordinary income, £11,104; ordinary expenditure, £11,481. Annual subscriptions have kept a good level during the last five years.

RATES OF SUBSCRIPTION: Twelve months, 6/6; Six months, 4/-; Three months, 2/-, post free to any address in the United Kingdom. Abroad, Twelve months, 8/8; Six months, 5/-; Three months, 2/6, post free.—Address The Subscription Dept., "THE HOSPITAL," The Hospital Building, 28/29 Southampton Street, Strand, London, W.C.

